# Factors influencing time management skills among nurses in North West Bank, Palestine

**DOI:** 10.1186/s12912-023-01560-x

**Published:** 2023-10-17

**Authors:** Raj’a Nayef Zyoud

**Affiliations:** Nursing Management and Administration, Faculty of Nursing, Arab American University/ Jenin- Palestine, 13 Zababdeh, P.O. Box 240, Jenin, Palestine

**Keywords:** Time management, Factors, Nurses, Organizing, Planning, Prioritizing activities

## Abstract

**Background:**

In today’s companies, time management abilities have grown as a significant predictor of nurses’ success. Organizations have simplified their internal operations and flattened their organizational structures in an effort to increase productivity and cut expenses. As a result, successful time management skills are particularly crucial for nurses in recently restructured healthcare organizations. This study aimed at exploring factors influencing time management skills among Palestinian nurses.

**Methods:**

Cross-sectional quantitative study of all nurses (715) working in private and government hospitals and primary healthcare centers in north Palestine was conducted. Time management skills were measured on a continuous scale using the Nursing Time Management Scale (NTMS), Arabic version. The scale measures various aspects of time management including goal setting, planning, scheduling, and organizing activities. The relationship between time management skills and background variables was assessed using the multivariate linear regression.

**Results:**

The average total score for NTMS scale was 63.39 out of a total score of 90. This score indicates relatively good time management skills among the respondents. The multivariate linear regression results showed that females obtained slightly lower scores than males, coefficient = -2.36, p = 0.043. Nurses in primary care centers had significantly higher scores than nurses who work at hospitals, coefficient = 4.47, p = 0.004. The type of healthcare organization emerged as a significant factor predicting time management skills. Nurses in private hospitals had worse time management skills than nurses in government hospitals, coefficient = -12.27, p < 0.001. Nurse supervisors had better time management skills than staff nurses, coefficient = 4.01, p = 0.023. Nurses working in non-teaching hospitals had worse time management skills than nurses in teaching hospitals, coefficient = − 3.86, p = 0.001. Nurses who did not attend a time management course had worse time management skills than nurses who attended time management course, coefficient = − 4.05, p = p < 0.001.

**Conclusions:**

Healthcare institutions should consider organizational and individual factors to improve the time management skills of their staff. Time management training interventions are proven and effective policies that are recommended to be adopted by all healthcare centers.

**Supplementary Information:**

The online version contains supplementary material available at 10.1186/s12912-023-01560-x.

## Background

Time is considered one of the most important resources [1]. Using time effectively can have enormous benefits for the individual and for the work environment. Building nurses’ time management skills can improve their well-being and quality of their work [2]. Overworked health professionals and workers’ burnout are related to poor quality of care provided to patients and unsafe care [1]. Good time management will not decrease the workload but will allow greater control over time, the workflow, and will reduce work stress [3]. Effective time management enables nurses to prioritize care, devote their valuable time and attention to the most critical duties first and delegate those that may be waited till later. However, good time management skills do not only assist nurses in prioritizing tasks but also in eliminating unnecessary tasks which would be considered as time wasters [4]. In addition to physical fatigue, shift work, and low decision autonomy, nurses report that shortage of time is one of the most important sources of work stress [4]. Lack of good time management skills can lead to anxiety, burnout, job dissatisfaction, and negative consequences to the whole organization, and finally to quality of patient care [5, 6].

Despite the importance of time management skills in the nursing profession, there is a lack of studies investigating those skills among nurses in the Palestinian context. Therefore, this study aims to explore factors related to time management skills among nurses in Palestine. The findings can be useful for nursing managers, policy makers, and national nursing directors to propose training programs to improve the quality of care. Good time management skills can reduce stress, improve psychological well-being, and increase work productivity. A quasi-experimental study of 60 nurses from all wards in a hospital in Tehran demonstrated improvements in psychological well-being and trust in staff after a one-day educational workshop on the importance of time management, practical strategies of managing time, and causes of wasted nursing time [2]. Another educational intervention of time management skills for head nurses was assessed through a self-administered questionnaire measuring four-time management skills (goal setting and prioritizing, time mechanic, time control, and organization). Results showed that nurses were applying these skills significantly more after the educational intervention, indicating that the educational program could have contributed to their improved performance [7]. Good time management skills can improve the quality of care provided for patients [1]. A pre- post intervention study on the impact of a time management educational pamphlet on nurses’ stress showed effectiveness of this self-learning module in reducing stress among acute care nurses in Egypt [3]. Stress levels as measured by the expanded nursing stress scale in (ENSS) where significantly reduced three months after the intervention. The educational pamphlets contained instructions for nurses on strategies about how to manage time effectively. According to research, effective strategies of time management include (1) Determining goals and objectives, (2) Prioritizing the goals and objectives [8], (3) Listing the activities that need to be done to achieve the goals, (4) Prioritizing the activities [8], (5) Understanding one’s knowledge, skills, and confidence in administrative and clinical work [9], (6) Working smarter not harder [10]. Working smarter includes knowing when and how to delegate tasks, knowing how to automate tasks, knowing how to set priorities, breaking down large tasks to smaller, completing smaller tasks first, and learning how to say no [1].

## Methods

### Design, sample, and setting

A quantitative cross-sectional study was conducted. Data were collected from the whole population of nurses (census sample of 715) working in governmental and private facilities (17 hospitals in the North of the West Bank of Palestine and several primary health care clinics). Data were collected between March and August 2019. From the beginning, it has been decided that any questionnaire with more than 80% complete responses is considered a non-missing questionnaire. Out of the 715 eligible participants, 53 questionnaires had few missing. By the assistance of a focal person in each facility, the researcher managed to return back all missing questionnaires to participants and achieved a full response rate of 100%.

Inclusion criteria for selection of study participants included all nurses working in hospitals and primary healthcare centers at study time. Exclusion criteria included nurses who were sick and off-duty, home-care nurses and nurses working in elderly care centers at time of data collection.

### Study instrument and data collection

Time management skills were assessed using the Nursing Time Management Scale (NTMS), Arabic version, the psychometric properties of which have been published previously based on several other validated scales such as the Assessment of Time Management Skills (ATMS) [11], the Time Organization and Participation Scale (TOPS) [12], and the Time Management Behavior Scale (TMBS) [13]. In this study, the researcher used the NTMS, Arabic version that showed good psychometric properties [14]. The scale contains 17 items and measures various aspects of time management including goal setting, planning, scheduling, and organizing activities. Response categories were on a 5-point scale: (1) *never* (2) *infrequently* (3) *Sometimes* (4) *frequently* (5) *always*. Total sum score was calculated ranging between a minimum of 17 points representing poor time management skills and a maximum of 90 points representing the best time management skill. Socio-demographic variables included gender, age, years of experience, residence, and educational level. Other variables were studied included work place, type of organization, current job position, teaching hospital / clinic, and attending management courses.

Data was gathered using the self-administered approach. The researcher contacted and met the nursing directors of each hospital and primary healthcare center. The researcher explained the objectives and importance of the study and agreed upon assigning a focal person in each facility to follow-up distributing, checking, and gathering all questionnaires. Then the researcher personally collected the completed questionnaires from the nursing directors. To ensure the data quality and accuracy during data collection, the researched attached with each questionnaire an explanatory letter clarifying the study purpose and objectives. The researcher kept her mobile number on the explanatory letter for responding to any possible inquiries or unclear question.

### Data analysis

Data were entered (by 2 experienced data entry personnel) and analyzed (by the researcher herself) using SPSS version 25. Descriptive statistics were performed using the frequencies, percentages, means, and standard deviations. Inferential statistics using the multivariate linear regression was used to adjust for effects of confounding variables and identify the main factors influencing the time management skills. Statistical significance was set at alpha less than 0.05.

### Ethical consideration

Approval to collect the data was obtained from the Palestinian Ministry of Health. The completion of the questionnaires was voluntary and did not result in harm if nurses chose not to participate. The data collected from the nurses was confidential and the questionnaires were kept in a secure place for research purposes only. Informed consent was obtained from all subjects. Ethical approval to conduct this study was obtained from the IRB of the American Arab University, Palestine IRB #13.P.E/22. All procedures were performed in accordance with the Declaration of Helsinki guidelines.

## Results

Background characteristics of the participants are shown in Table 1. About 65.2% of the nurses were females and 34.8 males. The majority were above 35 years of age (43.9%) and held a bachelor degree (57.3%), and live in city (49.2%). Almost 72.6% worked in hospitals and 27.4% in outpatient clinics. Most of participants worked in government institutions (75.1%) while 24.9% worked in private institutions. The majority of nurses (45.3%) had more than 10 years of job experience. Finally, a large percent of the participants (58.0%) attended a time management course.

**Table 1:** Time management score by background variables, N = 715

	n	%	Mean	P
**Gender**				
* Male*	249	34.8	63.75	0.555
* Female*	466	65.2	63.21	
**Age**				
* Less than*	201	28.1	61.10	**0.002**
* 25–35*	200	28.0	62.13	
* More than 35*	314	43.9	65.67	
**Residence**				
* City*	352	49.2	64.97	0.993
* Village*	300	42.0	62.27	
* Camp*	63	8.8	60.02	
**Educational level**				
* Technical*	271	37.9	62.25	0.077
* Bachelor*	410	57.3	63.76	
* Master or above*	34	4.8	68.18	
**Work Place**				
Hospital	519	72.6	61.76	**0.000**
Patient community clinic	196	27.4	67.74	
**Type of Organization**				
Government	537	75.1	66.86	**0.000**
Private	178	24.9	52.96	
**Current Job Position**				
Nurse	631	88.3	62.96	**0.038**
Nurse Supervisor	83	11.6	66.64	
**Job Experience**				
Less than 5 years	225	31.5	61.21	**0.002**
Between 5 10 years	165	23.1	62.33	
More than 10 years	324	45.3	65.61	
**Teaching hospital/clinic**				
Yes	396	55.4	65.27	**0.000**
No	319	44.6	61.06	
**Attended time management course**				
Yes	415	58.0	65.22	**0.000**
No	300	42.0	60.87	

Table 2 shows that the average total score for the NTMS was 63.39 out of a total score of 90. The highest NTMS score was for “I gather all equipment that will be needed before starting an activity” with mean 3.94, followed by “I document the nursing intervention as soon as possible after the activity is completed” with mean 3.93, and “I maintain a clean work area, and keep your desk organized” with mean 3.91. The lowest score was for " I spend enough time planning” with mean 3.37 followed by “I have a set of goals for the entire week” with mean 3.41 and " I force myself to make time for planning” with mean 3.51.

**Table 2:** The NTMS scale items on a range from 1 to 5, N = 715

	Mean	Frequencies				
		Never	Infrequently	Sometimes	Frequently	Always
I write a set of goals for myself for each day	3.53	12.2	9.94	22.69	23.25	31.9
I have a set of goals for the entire week	3.41	10.9	13.71	23.64	26.85	24.9
I force myself to make time for planning	3.51	9.09	12.45	24.2	26.99	27.3
I spend enough time planning	3.37	11.2	12.87	25.45	28.53	
I have a time to think about how plans will be translated into action	3.5	8.39	12.31	25.45	28.11	25.7
I have a clear idea of what you want to accomplish during day and make list of activities	3.62	7.55	9.37	25.45	28.81	28.8
Coordination of Medication Administration	3.84	4.76	7.14	20.73	33.47	33.9
Coordination of Treatments	3.84	5.6	9.24	15.69	34.03	35.4
Coordination of Procedures	3.86	4.91	8.7	17.81	32.12	36.5
Determine how reports will be given and received between shifts	3.87	5.04	9.8	15.41	32.35	37.4
I maintain a clean work area, and keep your desk organized	3.91	4.2	9.1	15.97	32.77	38
I group activities that are in the same location	3.82	4.62	9.24	20.45	30.39	35.3
I gather all equipment that will be needed before starting an activity.	3.94	4.62	7.7	15.41	33.19	39.1
I estimate the time needed to complete the task	3.85	3.92	8.96	17.93	36.13	33.1
I document the nursing intervention as soon as possible after the activity is completed	3.93	3.92	9.52	14.57	34.03	38
I handle paper work efficiently	3.86	5.18	8.68	16.95	33.33	35.9
I utilize appropriate technology to facilitate communication and documentation	3.78	7.01	7.99	19.07	31.98	33.9

Table 3 shows the results of the multivariate linear regression. Females obtained a slightly lower scores than males, coefficient = -2.36, p = 0.043. Nurses in primary care centers had significantly higher scores than nurses who work at hospitals, coefficient = 4.47, p = 0.004. The type of healthcare organization emerged as a significant factor predicting time management skills. Nurses in private hospitals had worse time management skills than nurses in government hospitals, coefficient = -12.27, p < 0.001.

Nurse supervisors had better time management skills than staff nurses, coefficient = 4.01, p = 0.023. Nurses working in non-teaching hospitals had worse time management skills than nurses in teaching hospitals, coefficient = − 3.86, p = 0.001. Nurses who did not attend a time management course had worse time management skills than nurses who attended time management course, coefficient = − 4.05, p = p < 0.001.

**Table 3:** Multivariate Time management score by background variables, N = 715

	Coef.	P	[95% Conf.	Interval
**Gender**				
Male				
Female	-2.36	**0.043**	-4.656	-0.072
**Age**				
Less than				
25–35	-0.21	0.886	-3.012	2.602
More than 35	1.65	0.323	-1.624	4.919
**Residence**				
City				
Village	-1.66	0.131	-3.817	0.498
Camp	-1.87	0.321	-5.572	1.831
**Educational level**				
Technical				
Bachelor	-1.39	0.217	-3.593	0.816
Master or above	0.84	0.745	-4.248	5.935
**Work Place**				
Hospital				
Patient community clinic	4.47	**0.004**	1.439	7.493
**Type of Organization**				
Government				
Private	-12.27	**0.000**	-14.892	-9.657
**Current Job Position**				
Nurse				
Nurse Supervisor	4.01	**0.023**	0.563	7.457
**Job Experience**				
Less than 5 years				
Between 5 10 years	0.22	0.883	-2.670	3.103
More than 10 years	-1.14	0.486	-4.357	2.075
**Teaching hospital/clinic**				
Yes				
No	-3.86	**0.001**	-6.093	-1.623
**Attended time management course**				
Yes				
No	-4.05	**0.000**	-6.278	-1.814

## Discussion

This study aimed at exploring factors that influence time management among nurses. The average total score for the whole sample was 63.39 out of a total score of 90. This score indicates relatively good time management skills among the respondents. This is consistent with previous research from Ethiopia [15], Nigeria [16], and Egypt [17]. Gender, workplace (clinic vs. hospital), job position, health center type (teaching vs. non-teaching), and attending a time management course were significantly related to time management skills while job experience, educational level, age, and place of residence were not. Male nurses in this study obtained a slightly higher time management score than females. The relationship between time management and gender in the literature is inconsistent. Some studies found that men have better time management skills [18, 19], others found that women are better in time management [20, 21], while others found no relationship between time management skills and gender [22]. Contrary to previous studies which found a positive correlation between time management skills and education, age, and job experience [21, 23], this paper did not find a relationship between time management skills and these variables. This could be because previous studies did not control for confounding variables through regression analyses. In this study, time management skills significantly increased with job experience, age, and education in the bivariate analyses but not in the adjusted multivariate analysis. The finding the senior nurses have better time management skills than junior nurses is consistent with all previous literature [21, 24]. This result indicate that seniority is more important in developing time management skills than age, education, or job experience. This could be because senior nurses usually enroll in management courses which could include time management aspects. Indeed, nurses who had attended a management course were significantly more likely to possess excellent time management skills than those who did not.

Our finding points out that attending a time management course significantly improves time management skills is consistent with numerous previous studies which showed that time management interventions did not only improve time management skills but also job satisfaction [25], psychological well-being [2] and decrease work stress [26, 27]. This finding is in line with previous reports indicating that the effectiveness of time management interventions can also be modified by Contextual factors such as organizational policies and personal motivation [28]. Nurses who work in private health institutions scored significantly lower on the time management scale than nurses who work in government clinics. Government hospitals and clinics in Palestine are usually affordable and as a result more crowded than private hospitals. The overcrowding could be a motivation for nurses to develop their time management skills in order to cope with their work overload. Contrary to our finding, a study in Pakistan found that staff shortages, burnout, and working under pressure negatively affects nurses’ time management behaviors [28]. These contradictory results could be because of different contextual factors. Nursing and hospital policies may vary between countries.

On the other hand, other studies showed that more crowded hospitals provide better quality services probably because healthcare professionals gain more experience due to a busier work environment [29]). Another explanation for worse time management skills in private healthcare centers could be related to different organizational policies between governmental and private health centers. Government health care centers in Palestine usually offer more staff development programs for employees compared to private hospitals. For example, government hospitals provide more training opportunities, educational scholarships, study days, attending free national and international workshops and conferences. A mixed methods analysis of factors related to time management among health workers in Ethiopia showed that organizational factors and policies such as performance appraisal, the benefit system, and compensations are among the most significant factors influencing time management behaviors [15]. Several studies have also demonstrated that organizational policies are strongly related to staff satisfaction and time management skills in Palestine [30], Nigeria [16], and Egypt [17].

### Limitations

The cross-sectional nature of this study limits causal inferences. In addition, self-reporting may bias responses of study participants. However, the validity of the questionnaire has been proven in previous studies [13, 31].

### Implications for nursing

Nurses would benefit tremendously from improving their time management skills. Therefore, nurse managers should facilitate ongoing time management training for nursing staff at hospitals and primary healthcare centers. Government health care centers in Palestine usually offer more staff development programs for employees compared to private hospitals. For example, government hospitals provide more training opportunities, educational scholarships, study days, attending free national and international workshops and conferences. This study revealed that there was a striking weakness in the management skills among nurses especially working in private hospitals compared to government hospitals. Therefore, it is highly recommended that private hospitals attention should be directed to this issue and take remedial actions such as conducting training sessions or developing policies to address this problem. Given the dearth of research of time management skills specifically in the nursing field, researches should conduct more studies on the subject to investigate the reasons behind this difference between government and private healthcare provider hospitals and primary healthcare centers and clinics. More research also needed on this topic within the Palestinian context. Other aspects of time management skills should also be investigated such as strategies in setting priorities, avoiding time wasting, and reducing work load.

## Conclusion

Time management skills are important for nurses and their profession. Healthcare institutions should consider organizational and individual factors to improve the time management skills of their staff which consequently reflect positively on the whole organization. Time management training interventions are proven and effective policies that are recommended to be adopted by all health care centers.

### Electronic supplementary material

Below is the link to the electronic supplementary material.

Supplementary Material 1

## Data Availability

The data sets used and analyzed during the current study available from the corresponding author on reasonable request.
