# Supplementary material for: Factors influencing time management skills among nurses in North West Bank, Palestine
Source: BMC Nurs. 2023 Oct 17;22:386. doi: 10.1186/s12912-023-01560-x (PMC10580556; doi:10.1186/s12912-023-01560-x)
Supplement: Supplementary file 1 — Supplementary Material 1 [file 12912_2023_1560_MOESM1_ESM.pdf]

The NTMS scale

	Never	Infrequently	Sometimes	Frequently	Always
I write a set of goals for myself for each day					
I have a set of goals for the entire week					
I force myself to make time for planning					
I spend enough time planning					
I have a time to think about how plans will be translated into action					
I have a clear idea of what you want to accomplish during day and make list of activities					
Coordination of Medication Administration					
Coordination of Treatments					
Coordination of Procedures					
Determine how reports will be given and received between shifts					
I maintain a clean work area, and keep your desk organized					
I group activities that are in the same location					
I gather all equipment that will be needed before starting an activity.					
I estimate the time needed to complete the task					
I document the nursing intervention as soon as possible after the activity is completed					
I handle paper work efficiently					
I utilize appropriate technology to facilitate communication and documentation					
